# Temporomandibular Disorders: Current Concepts and Controversies in Diagnosis and Management

**DOI:** 10.3390/diagnostics11030459

**Published:** 2021-03-06

**Authors:** Dion Tik Shun Li, Yiu Yan Leung

**Affiliations:** Department of Oral and Maxillofacial Surgery, Faculty of Dentistry, The University of Hong Kong, Hong Kong, China; diontsli@hku.hk

**Keywords:** temporomandibular disorders, temporomandibular joint, TMD, facial pain, craniomandibular disorders

## Abstract

Temporomandibular disorders (TMD) are a group of orofacial pain conditions which are the most common non-dental pain complaint in the maxillofacial region. Due to the complexity of the etiology, the diagnosis and management of TMD remain a challenge where consensus is still lacking in many aspects. While clinical examination is considered the most important process in the diagnosis of TMD, imaging may serve as a valuable adjunct in selected cases. Depending on the type of TMD, many treatment modalities have been proposed, ranging from conservative options to open surgical procedures. In this review, the authors discuss the present thinking in the etiology and classification of TMD, followed by the diagnostic approach and the current trend and controversies in management.

## 1. Introduction

The diagnosis and management of the most common cause of non-dental pain in the maxillofacial region, namely temporomandibular disorders (TMD), remains a challenge for clinicians to this day, despite extensive clinical research into the topic. This is because TMD is a broad term comprising of different conditions with complex etiologies, with symptoms that vary in intensity. Intriguingly, some signs and symptoms resolve spontaneously even without treatment, whereas others persist for years despite all treatment options having been exhausted. More perplexing is that while some may have a recognizable physical basis, many cases of TMD also involve a significant biopsychosocial component [1,2,3] with various associated psychological symptoms, such as depression and anxiety [4,5,6]. Numerous treatment modalities have been proposed over the years, with some becoming obsolete while others are gaining in popularity. Nevertheless, it seems that there is no single solution for every case as many different symptoms are included in TMD. Controversies exist in the literature regarding the diagnosis and the management protocol for TMD, hence the selection of treatment modality may often be largely influenced by the expertise of the treating healthcare provider.

In general, TMD is believed to affect anywhere between 5 and 15% of adults in the population [7,8,9,10], yet TMD related symptoms have been reported to be present in up to 50% of adults [11]. Interestingly, there is evidence that the prevalence of TMD appears to be on the rise in recent years [12,13,14,15,16]. A recent systematic review and meta-analysis in 2021 concluded that the prevalence of TMD was 31% for adults and 11% for children and adolescence [17]. The fact that TMD encompasses a broad assortment of clinical diseases is partially responsible for the wide range of prevalence rate estimates among studies, as the classification of different types of TMD, the distinction between disease and non-disease, as well as whether to include those with inactive disease as having TMD, may all be subject to the partialities of the assessing clinical researchers. In addition, studies that are questionnaire-based might over-estimate the prevalence of TMD, as the symptoms of many other conditions, such as headache not caused by TMD, dental pain, neuropathic conditions, and otological diseases, can mimic the presentation of TMD.

TMD represents a significant and complex health problem, with opinions regarding the appropriate course of management often equivocal. In this review, we discuss the current concepts in the etiology and diagnosis of TMD, followed by an up-to-date management approach from a surgeons’ perspective.

## 2. Etiologies and Classifications

As an umbrella term for pain and dysfunction of the temporomandibular regions, TMD encompasses a wide variety of clinical conditions. The etiologies of TMD are multi-factorial and can be attributed to both physical and psychosocial factors [18,19,20]. The physical causes can broadly be divided into arthrogenous, and the more common myogenous origins. Many believe that TMD symptoms of arthrogenous origin may be related to internal derangement of the TMJ, which can be defined as a disruption of the internal aspect of the joint, and usually pertains to an articular disc that has been displaced. Although internal derangement does not necessarily lead to pain, it is generally believed that internal derangement precedes degenerative joint diseases, namely osteoarthritis [21]. Osteoarthritis is associated with pain and functional impairment of the TMJ, and is characterized by subchondral bony changes such as cortical erosion and marginal lipping, secondary to pathological changes of the cartilaginous articular disc [22]. Note that the term “osteoarthrosis” has been used as a synonym of osteoarthritis, but also has been used to describe degenerative joint changes of non-inflammatory cause [22]. The severity of internal derangement has been classified by Wilkes into five stages with relations to pain, mouth opening, disc location and anatomy [21]. The classification ranges from painless clicking of the joint (Stage I) to severe pain of the joint with severe degenerative bony changes (Stage V), which has served as an aid to guide treatment options in the management of arthrogenous TMD.

While structural anomalies of the TMJ may predispose the patients to symptoms of TMD [23], it should be noted that not all those with structural abnormalities suffer from the same level of clinical symptoms. Apart from physical causes, the association between biopsychosocial factors and TMD has been described by many [1,2,3,4,19,24]. Similar to other chronic pain conditions, such as back pain and headache, it appears that there are those in the population who are at risk for developing symptomatic TMD, who also share a certain psychological profile and dysfunction [25,26]. Higher levels of depression and somatization are associated with TMD of arthrogenous and myogenous origins [27]. Moreover, in those with pre-existing TMD, symptoms may be exacerbated during times of stressful events. For example, recent studies have suggested that the during periods of lockdown and social isolation due to the ongoing COVID-19 pandemic, an impact was found on the prevalence of depressive symptoms, stress, as well as pain related to TMD [28,29]. The finding that psychological variables are closely tied to the development of TMD has been confirmed by the Orofacial Pain: Prospective Evaluation and Risk Assessment (OPPERA) study, which found that TMD onset was strongly associated with somatic symptoms, while previous life events, perceived stress and negative affect were also associated with the incidence of TMD [30].

What makes the diagnosis and classification of TMD complicated at times is that many patients present with multiple diagnoses of TMD simultaneously, and it is impossible to isolate the condition to a single particular cause. When discussing about TMD, most clinical researchers refer to those pain conditions that are most commonly seen. However, one must not forget that disorders related to the TMJ include those that are less routinely encountered. Importantly, the presentation of these uncommon conditions of the TMJ may initially mimic those of the more common TMD, yet the management approach may be completely different. For example, a patient who presents with ankylosis of the TMJ may initially present with signs and symptoms similar to closed-lock due to disc displacement, but the standard treatment for ankylosis is surgical release of ankylosis, while conservative or minimally invasive options, such as arthrocentesis, are usually indicated for closed-lock of the TMJ due to disc displacement.

The crude classification of the most common diagnoses of TMD into arthrogenous, myogenous, or of mixed origin is helpful in steering the clinician into the appropriate path in the initial phases of management. However, more specific diagnoses are usually required, especially if the management progresses beyond conservative options. In the past, classification was often confusing, with many different terminologies referring to similar entities. Today, the Diagnostic Criteria for Temporomandibular Disorders (DC/TMD) is the most widely accepted and standardized tool for assessment and classification of TMD, with sensitivity and specificity established for the most common diagnoses of TMD [31]. Recognizing that TMD contains a structural as well as a biopsychosocial component, the DC/TMD consists of two Axes in its assessment. Axis-I contains a protocol for a prescribed physical examination to arrive at specific physical diagnoses of TMD with regard to the joint and musculature, while Axis-II contains several instruments to assess the psychological state of the patient.

There are 12 most common diagnoses of TMD described in Axis-I of the DC/TMD, which are divided into painful conditions (myalgia, local myalgia, myofascial pain, myofascial pain with referral, arthralgia, headache attributed to TMD) and non-painful conditions (disc displacement with reduction, disc displacement with reduction with intermittent locking, disc displacement without reduction with limited opening, disc displacement without reduction without limited opening, degenerative joint disease, subluxation) [31] (Table 1). Note that in many cases, multiple diagnoses are present at any timepoint in a single patient, and that diagnoses may change as the disease progresses or resolves. For example, a patient with complaints of joint clicking with pain in the TMJ and masseter muscle, and headache during mouth opening may be diagnosed with having local myalgia, arthralgia, disc displacement with reduction, and headache attributed to TMD. The classification of TMD also includes those that are less common, but clinically important diseases [32]. Some of these less common diagnoses include fractures of the TMJ, manifestations of systemic diseases, as well as rare conditions such as neoplasms and developmental disorders (Table 2) [32]. However, when these diagnoses do not fit the clinical symptoms, other conditions should also be considered.

## 3. Diagnostic Approach

The signs and symptoms of TMD may mimic other orofacial pain conditions. Although precise physical diagnosis into the type of TMD is helpful in developing an appropriate treatment plan, it might not be straight forward in every case. Taking a patients’ history is an important part of diagnosing the TMJ condition. The acquisition of history follows the usual format. Apart from the chief complaint, inquiries should be made regarding any history of trauma or previous episodes, aggravating factors, such as eating, talking, yawning or spontaneous background pain, and any previous investigations or treatment. The severity of pain should also be graded using a visual analogue scale (VAS), so treatment progress can be quantitatively monitored. A past and current medical history, including a full medications list, may reveal any comorbidities that may be related to TMD. The clinician should note any habits such as smoking, drinking and recreational drug use, and any history of clenching or bruxism as complained by the patients’ bed partner. Additionally, the clinician should ask questions regarding stress and level of life satisfaction, and whether there are any recent life events, such as change of job or loss of a loved one. Although most clinicians treating TMD may be experienced with acquiring a clinical history, some may not be comfortable with taking a psychological history. If desired, the clinician may employ the numerous psychosocial instruments available to aid in their diagnosis, such as those in Axis-II of DC/TMD [31]. When necessary, the patient may be referred for a psychological assessment.

Most clinicians who treat orofacial pain believe clinical examination is the most crucial process of diagnosing TMD. The location of pain, and whether the pain is localized, remains within or spreads beyond the confines of the muscle, should be confirmed with palpation, which is done at rest and during mandibular function. Clicking or crepitus upon mandibular function might be quite obvious in some cases, and the detection might be aided by the use of a stethoscope. Intriguingly, the presence or location of clicking detected by the clinician might be different from that reported by the patient, and this should be documented. The range of mouth opening measured should include pain-free maximum mouth opening, maximum unassisted mouth opening, and maximum assisted mouth opening. Any deviation of the mandible may indicate differential obstruction of the movement of the mandibular condyle in rotation and/or translation. An intra-oral examination is performed to rule out any mucosal pathologies of the oral cavity and oropharyngeal region, as well as to assess the state of the dentition.

### 3.1. Imaging and Other Investigations

**Figure 1:**
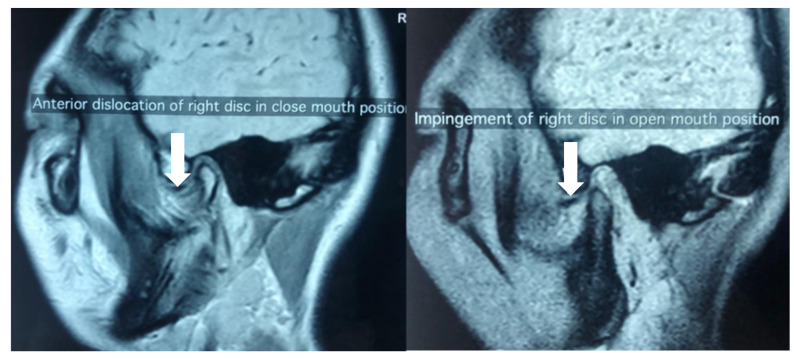
Magnetic resonance imaging (MRI) showing anteriorly displaced disc in both the close and open mouth position in a patient presented with lock jaw.

Imaging is considered to be a useful adjunct in the diagnosis of TMD. Although the diagnostic information provided by plain radiographs like orthopantomogram is limited, they are convenient, simple and serve to rule out some of the differential diagnoses of the bony TMJ, such as fractures, ankylosis, growth disturbances, as well as neoplasms. For the most common types of TMD which clinical presentation is typical, many units might not routinely employ additional imaging. This is due to availability and cost, and that additional imaging might not alter the initial management plan. However, when further information is desired, magnetic resonance imaging (MRI) is the gold standard for TMJ imaging, and is useful in assessing the status of the osseous, as well as the non-osseous structures of the TMJ, such as the masticatory muscles, ligaments and the cartilaginous disc [33] (Figure 1). Classification systems, such as Wilkes [21], combine clinical and MRI findings to stage the extent of internal derangement in order to guide treatment protocol. MRI is therefore considered mandatory prior to any surgical intervention.

While MRI is the most commonly used diagnostic imaging for the common diagnoses of TMD, other imaging modalities are also employed for specific indications. Cone-beam computed tomography (CBCT) has been used to further assess the osseous structure of the TMJ [34,35,36]. This may be desirable in cases of TMJ ankylosis, benign bony neoplasms or overgrowth, or for the planning of osseous surgery, such as for eminectomy for recurrence TMJ dislocation. However, for most other diagnoses of TMJ, the value of CBCT is not well-established since the information provided in terms of soft tissues is limited [36]. Moreover, the use of ultrasound as a diagnostic tool for TMD has been suggested [15,37,38]. Ultrasound has the advantages of being non-invasive, cheap, and widely available in many health institutions, yet the effectiveness as a diagnostic method remains to be confirmed [15]. For some inflammatory conditions of the TMJ, such as osteoarthritis and joint inflammation, bone scintigraphy may be of value as a diagnostic tool [39,40,41,42,43]. Moreover, bone scintigraphy has been proposed as a method for the evaluation of active TMJ condylar growth, but it has been shown that both the sensitivity and specificity are low for this indication [44].

Apart from the different imaging modalities available, other investigations are not commonly done for most diagnoses of TMD, except in specific indications. For example, blood investigations may be done for TMD related to systemic conditions, such as rheumatoid arthritis or gout. In the case of uncertain diagnoses of rare diseases or neoplasms, tissue biopsies might be taken, which may be done by fine-needle aspiration, arthroscopic or open joint approach.

### 3.2. Diagnosis of TMD

Recognizing the causes of pain and dysfunction related to TMD is important in order to guide treatment decisions. For instance, different treatment options are often employed for the treatment of myogenous versus arthrogenous TMD. Moreover, in those patients who present with TMD symptoms without an obvious physical cause, who also suffer from psychological comorbidities, may be best treated by counselling and psychological intervention.

The most important part of the diagnosis of TMD is to differentiate the common diseases from those clinically significant, but unusual conditions, as well as conditions that are more serious which urgent attention is needed. For example, some neoplasms, such as chondrosarcoma of the TMJ may initially share signs and symptoms as some of the common diagnoses of TMD, such as pain at the preauricular region and limited opening. Another example that requires urgent attention is temporal arteritis, which is an inflammatory condition of the temporal vessels with some TMD-like symptoms, such as headache, pain in the temporal region, and limited mouth opening. However, temporal arteritis is a medical emergency which may cause permanent blindness if not treated promptly. Some of the differential diagnoses of orofacial pain that may mimic TMD are listed in Table 3 [45].

## 4. Treatment Modalities—A Change in Paradigm?

The goals of treatment for TMD include reduction of pain and improvement of jaw function. Additionally, treatment with the goal of behavioural change may be important in the reduction of tension and parafunction. Currently, physically restoring the disc position in the case of internal derangement is not the primary treatment objective as it may not be relevant to clinical improvement [46,47], unless of course if there is inflammation related to disc displacement then it should be addressed. Symptoms of TMD should be addressed promptly, as chronic pain becomes more difficult to manage due to psychological deterioration and somatization [2,19]. Since conservative options are less likely to cause any harm, they are usually indicated in the early stages of treatment. This is especially true when definitive diagnosis is difficult to ascertain and treatment is performed empirically. However, there is no agreement on how long conservative treatment should be attempted before progressing to other options when clear benefits are not observed. Although the treatment of TMD has shifted away from open procedures which were once popular, the demonstrated success of minimally invasive options may indicate that they may be considered as an early option for those cases refractory to conservatory approaches.

### 4.1. Conservative Options

**Figure 2:**
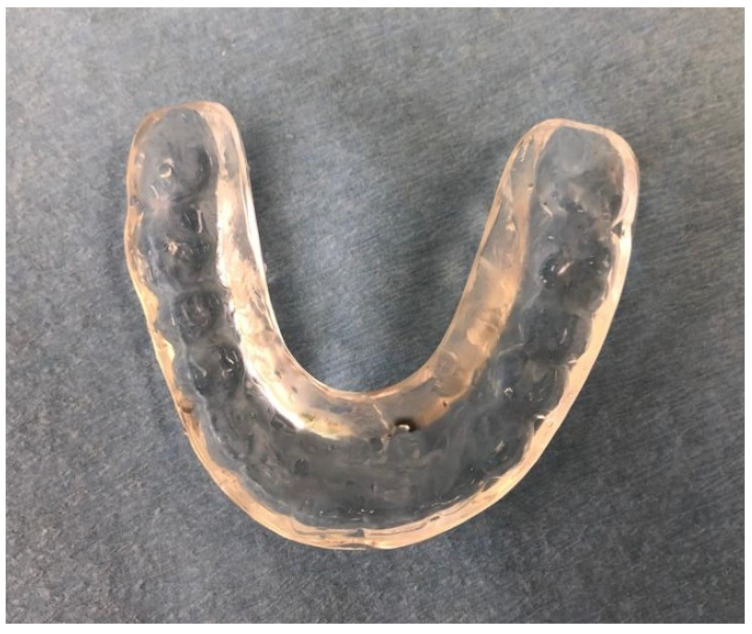
Occlusal splint for the management of temporomandibular disorders (TMD) and bruxism.

The initial management of TMD may include various medications, such as analgesics, non-steroidal anti-inflammatory drugs (NSAIDs), anxiolytics, and anti-depressants. Occlusal appliances of various designs are routinely prescribed, which represent a non-invasive option with minimal risks (Figure 2). The use of occlusal splint therapy has been shown to reduce pain intensity and increase maximal mouth opening [48]. However, whether the effect of an occlusal splint is due to the placebo effect has been questioned, and that the evidence of its efficacy remains to be low [49,50]. A systematic review in 2018 by Alkhutari et al. has suggested that the use of occlusal splint may improve patient-centred treatment outcomes, which may be more than merely a placebo effect [51]. Multiple designs are available, such as hard, soft, and anterior repositioning splint. At present, there is no consensus on which design is superior, as results from different studies are equivocal in terms of the efficacy of different designs of occlusal splints [50,52].

Physiotherapy has been suggested to be an important part in the management of TMD [53,54], which may be particularly useful for myalgia or myofascial pain. Understanding the loading of the stomatognathic system, and the existence of any tension and parafunctions, is important in delivering physiotherapy such as muscle training and changing of behaviour. Evidence shows that physiotherapy is effective in treatment of TMD, in particular the headache symptoms associated with the condition; future research into this area will further ascertain these findings [54]. For myogenous TMD, Botox injection and dry-needling techniques have been suggested [55,56]. Note that Botox is not considered a standard treatment option for TMD, while dry-needling, or acupuncture, may be an effective method to reduce tension in some patients. Additionally, initial results regarding extracorporeal shock wave therapy for myogenous TMD appear to show positive results [57,58].

There has been increasing evidence demonstrating that psychosocial assessment serves as a powerful tool in terms of predicting treatment outcome [59,60]. For those patients with a significant psychosocial component, counselling seems to be a promising treatment adjunct [50,61,62,63], which might be most beneficial when included in a multimodal approach [50]. Other conservative treatment options for TMD include stress reduction techniques and diet modification. In the past, a causative relationship between occlusion and TMD had been suggested, but it is now considered an outdated theory not supported by robust evidence, and occlusal adjustment is an irreversible treatment which is no longer supported by the recent literature [64,65,66,67].

### 4.2. Minimally Invasive Options—Arthroscopy, Arthrocentesis and Intra-Articular Injections

In the 1980s, the availability of MRI has led clinicians to acknowledge the structural anomalies related to TMD. This has resulted in a boom of open joint surgeries, which were unfortunately ineffective in the most part. For those cases of TMD that are arthrogenous and not responsive to conservative treatment, more focus has since been shifted to minimally invasive procedures which have shown promising clinical results.

**Figure 3:**
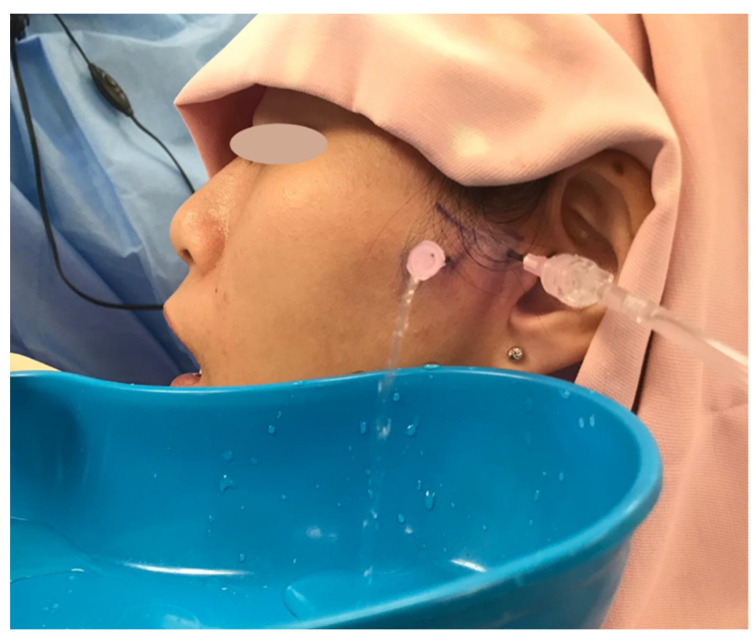
Arthrocentesis performed under local anaesthesia.

Arthroscopy of the TMJ was initially pioneered by the Japanese in the 1970s [68,69], and later popularized by the Americans [70,71,72]. TMJ arthroscopy may involve lysis and lavage of the superior joint space, as well as operative procedures, such as repositioning of a displaced disc, arthroplasty, and removal of inflamed tissues and adhesions. The efficacy of arthroscopy has since been well-recognized [73,74,75,76,77,78,79], and has been found that the therapeutic effect was mainly due to lysis and lavage but not disc position [80]. It was due to this finding that a modification was made, where lysis and lavage was performed without arthroscopic view. This was termed arthrocentesis which was first described by Nitzan et al., in 1991 [81], with efficacy that has since been well-documented [46,82,83,84,85,86,87,88,89,90,91,92,93,94] (Figure 3).

In addition to the shift from open joint surgery to minimally invasive treatment for those cases not responsive to conservative treatment, recent literature seems to support that minimally invasive options may be attempted early for arthrogenous TMD [95,96], and this may represent a paradigm shift in the management protocol. A recent integrated review and meta-analysis performed by the authors of this article showed that arthrocentesis was beneficial, whether it was performed as an initial treatment, as an early or late treatment with regard to conservative treatment [97]. However, the best timing to perform arthrocentesis is still unclear due to the paucity of research on the topic, which warrants more future well-designed clinical trials [97].

Although both arthroscopy and arthrocentesis have been shown to be beneficial in the treatment of TMD, it is unclear which method produces better clinical results. In a systematic review and meta-analysis by Al-Moraissi, it was revealed that arthroscopy was superior to arthrocentesis in pain reduction and jaw function improvement, with similar complication rates for both methods [78]. However, other studies have shown comparable results with the two procedures [98,99]. Nevertheless, arthrocentesis has been suggested to be attempted first due to simplicity and cost-effectiveness, with a similar or potentially lower complication rate [99].

Several modifications have been suggested for the conventional arthrocentesis, which involves two puncture needles into the superior joint space guided by landmarks in relations to adjacent structures, followed by lavage with an irrigation solution. For example, single-puncture techniques employ specially designed devices, and may have both the inflow and outflow fluid going through a single cannula but with different ports. Although single-puncture techniques may appear more simple than double-puncture arthrocentesis, most studies to date have shown a similar clinical outcome between the two techniques [83,100,101,102]. In addition, ultrasound-guided arthrocentesis has been proposed to increase the accuracy of puncture into the superior joint space [103,104,105,106]. However, a recent systematic review by Leung et al. has shown that no additional benefit is seen with ultrasound-guided arthrocentesis compared to conventional arthrocentesis [107]. Furthermore, different pharmacological agents for intra-articular injection have been proposed, with the common ones including hyaluronic acid, corticosteroid, analgesics, and platelet-rich plasma [93,96,108,109]. Although promising results are seen in some studies, there is currently no consensus regarding which intra-articular injection agent is superior over the others.

Despite the reported efficacy, arthroscopy is seldom required in TMD patients, even in cases of true arthrogenous disorders. Additionally, arthrocentesis is still considered to be a controversial procedure [87], despite the documented efficacy and low complication rates. The reasons for this controversy are as follows. Firstly, some cases of TMD improve with mere conservative options, or even without treatment. Additionally, many cases of TMD are due to multiple etiologies, which may require a multimodal approach before any clear clinical improvement can be appreciated. In addition, intra-articular injection of corticosteroids is a simple and very effective treatment, which may be attempted prior to arthrocentesis. In short, minimally invasive procedures may be the answer in those patients with true arthrogenous TMD not responsive to conservative treatment options, whose condition also lack a significant biopsychosocial component.

### 4.3. Open Joint Surgery

Open surgical treatment for TMD is now uncommon, and is reserved for specific indications as well as end-stage diseases. Though, surgery may be the only viable option in some conditions, such as ankylosis and neoplasms, which require release of ankylosis and removal of tumour, respectively. Pending on the availability of equipment and skills, there is now an option of arthroscopic surgery for procedures that were only performed with an open-joint approach in the past. These procedures include disc repositioning procedures, removal of osteophyte, removal of pathologic tissue, and biopsy of the TMJ. In recent years, much work has been done regarding replacement of the TMJ with alloplastic prosthesis [110,111,112,113,114,115,116] with an observed improvement in prognosis and longevity. Due to this success, it is likely that we will see a continuous increase in popularity of alloplastic replacement of the TMJ for conditions such as end stage arthritic conditions, ankylosis, post-tumour resection, and developmental anomalies of the TMJ.

## 5. Conclusions

TMD represents a divergent group of orofacial pain symptoms which shares similarities with other chronic pain conditions. The etiology of TMD is often multi-factorial, and precise causes for the symptoms may be difficult to pinpoint. In the past, focus has been placed on the physical origins of TMD, but an at least equally significant psychosocial factor is now well-recognized. Consequently, a multimodal approach, which might include counselling and psychological therapy, is being increasingly advocated. Most instances of TMD are managed conservatively and empirically during the early phases of treatment, yet lingering in the conservative phase for an extended period when clinical improvement is unclear is not recommended. Though open joint surgery is rare nowadays and is reserved for specific situations, we may be in the midst of a changing paradigm which favours early minimally invasive procedures.

## Figures and Tables

**Table 1:** Common diagnoses of temporomandibular disorders (TMD) and their clinical findings.

**Painful Conditions**	**Clinical Findings**
Myalgia	Familiar pain in the masseter or temporalis upon palpation or mouth opening
Local Myalgia	Familiar pain in the masseter or temporalis localized to the site of palpation
Myofascial pain	Pain in the masseter or temporalis spreading beyond the site of palpation but within the confines of the muscle
Myofascial pain with referral	Pain in the masseter or temporalis beyond the confines of the muscle being palpated
Arthralgia	Familiar pain in the TMJ upon palpation or during function
Headache attributed to TMD	Headache in the temple upon palpation of the temporalis muscle or during function
**Non-Painful Conditions**	**Clinical Findings**
Disc displacement with reduction	Clicking in the TMJ upon function
Disc displacement with reduction with intermittent locking	Clicking in the TMJ with reported episodes of limited mouth opening
Disc displacement without reduction with limited opening	Limited mouth opening affecting function, with maximum assisted opening < 40mm
Disc displacement without reduction without limited opening	Limited mouth opening affecting function, with maximum assisted opening of ≥ 40mm
Degenerative joint disease	Crepitus of the TMJ upon function
Subluxation	History of jaw locking in an open mouth position, cannot close without a self-maneuver

Modified from Schiffman et al., 2014 [31].

**Table 2:** Some less common diagnoses of temporomandibular disorders (TMD).

**I. TMJ**
A. Joint pain
1. Arthritis
B. Joint disorders
1. Hypomobility disorders other than disc disorders
a. Adhesions/Adherence
b. Ankylosis (Fibrous or Osseous)
2. TMJ dislocations
C. Joint diseases
1. Systemic arthritides
2. Condylysis/Idiopathic condylar resorption
3. Osteochondritis dissecans
4. Osteonecrosis
5. Neoplasm
6. Synovial Chondromatosis
D. Fractures
E. Congenital/Developmental disorders
1. Aplasia
2. Hypoplasia
3. Hyperplasia
**II. Masticatory Muscles**
A. Muscle pain
1. Tendonitis
2. Myositis
3. Spasm
B. Contracture
C. Hypertrophy
D. Neoplasm
E. Movement Disorders
1. Orofacial dyskinesia
2. Oromandibular dystonia
F. Masticatory muscle pain related to central/systemic pain disorder
1. Fibromyalgia/widespread pain
**III. Associated Structures**
A. Coronoid hyperplasia

Modified from Peck et al., 2014 [32].

**Table 3:** Differential diagnosis of temporomandibular disorders (TMD)**.**

**Neuropathic Pain**
Trigeminal neuralgia
Glossopharyngeal neuralgia
Postherpetic neuralgia
Traumatic neuralgia
Burning mouth syndrome
Atypical odontalgia
Atypical facial pain
**Odontogenic Pain**
Dental caries
Periodontal disease
Dental abscess
Dental sensitivity
Cracked tooth syndrome
Periocoronitis
**Intracranial Pain**
Tumours
Aneurysms
Bleeding
Infection
**Pain from Other Adjacent Structures**
Ear
Nose
Throat
Eyes
Sinus
Salivary glands
Lymph nodes
Vasculature
Cervical region
**Headaches not Attributed to TMD**
Migraine
Cluster headache
Tension-type headache
Temporal arteritis
**Referred Pain**
**Psychogenic Pain**

Modified from Kumar et al. (2013) [45].
